# Evaluation of auto-segmentation accuracy of cloud-based artificial intelligence and atlas-based models

**DOI:** 10.1186/s13014-021-01896-1

**Published:** 2021-09-09

**Authors:** Yuka Urago, Hiroyuki Okamoto, Tomoya Kaneda, Naoya Murakami, Tairo Kashihara, Mihiro Takemori, Hiroki Nakayama, Kotaro Iijima, Takahito Chiba, Junichi Kuwahara, Shouichi Katsuta, Satoshi Nakamura, Weishan Chang, Hidetoshi Saitoh, Hiroshi Igaki

**Affiliations:** 1 Department of Radiological Sciences, Graduate School of Human Health Sciences, Tokyo Metropolitan University, 7-2-10 Higashi-Ogu, Arakawa-ku, Tokyo, 116-8551 Japan; 2 Department of Medical Physics, National Cancer Center Hospital, 5-1-1 Tsukiji, Chuo-ku, Tokyo, 104-0045 Japan; 3 Department of Radiation Oncology, National Cancer Center Hospital, 5-1-1 Tsukiji, Chuo-ku, Tokyo, 104-0045 Japan; 4 Department of Radiological Technology, National Cancer Center Hospital, 5-1-1 Tsukiji, Chuo-ku, Tokyo, 104-0045 Japan

**Keywords:** Artificial intelligence, Automatic segmentation, Deep learning neural network, Prostate cancer, Head and neck cancer

## Abstract

**Background:**

Contour delineation, a crucial process in radiation oncology, is time-consuming and inaccurate due to inter-observer variation has been a critical issue in this process. An atlas-based automatic segmentation was developed to improve the delineation efficiency and reduce inter-observer variation. Additionally, automated segmentation using artificial intelligence (AI) has recently become available. In this study, auto-segmentations by atlas- and AI-based models for Organs at Risk (OAR) in patients with prostate and head and neck cancer were performed and delineation accuracies were evaluated.

**Methods:**

Twenty-one patients with prostate cancer and 30 patients with head and neck cancer were evaluated. MIM Maestro was used to apply the atlas-based segmentation. MIM Contour ProtégéAI was used to apply the AI-based segmentation. Three similarity indices, the Dice similarity coefficient (DSC), Hausdorff distance (HD), and mean distance to agreement (MDA), were evaluated and compared with manual delineations. In addition, radiation oncologists visually evaluated the delineation accuracies.

**Results:**

Among patients with prostate cancer, the AI-based model demonstrated higher accuracy than the atlas-based on DSC, HD, and MDA for the bladder and rectum. Upon visual evaluation, some errors were observed in the atlas-based delineations when the boundary between the small bowel or the seminal vesicle and the bladder was unclear. For patients with head and neck cancer, no significant differences were observed between the two models for almost all OARs, except small delineations such as the optic chiasm and optic nerve. The DSC tended to be lower when the HD and the MDA were smaller in small volume delineations.

**Conclusions:**

In terms of efficiency, the processing time for head and neck cancers was much shorter than manual delineation. While quantitative evaluation with AI-based segmentation was significantly more accurate than atlas-based for prostate cancer, there was no significant difference for head and neck cancer. According to the results of visual evaluation, less necessity of manual correction in AI-based segmentation indicates that the segmentation efficiency of AI-based model is higher than that of atlas-based model. The effectiveness of the AI-based model can be expected to improve the segmentation efficiency and to significantly shorten the delineation time.

**Supplementary Information:**

The online version contains supplementary material available at 10.1186/s13014-021-01896-1.

## Background

In radiotherapy, radiation oncologists define the tumor and Organs at Risk (OARs) on computed tomography (CT) images in the treatment planning system (TPS) and their definitions are crucial to identify the radiotherapy region for the assessment of therapeutic outcomes and expected occurrence of toxicities. However, this process is generally time-consuming and burdensome, given the need for manual delineations. For example, in head and neck cancers treated with intensity-modulated radiotherapy (IMRT), the precise work of segmentation requires approximately 3 h [1], and it takes several days for the therapy regimen in addition to performing daily clinical work. Additionally, several studies have demonstrated that there is a significant deviation among experts [2–4]. Large inter-observer variation may cause inaccuracy in treatment. To standardize delineation and improve contouring efficiency in radiotherapy, automatic segmentation methods have been developed.

Atlas-based segmentation have been developed to improve contouring efficiency, which is expected to reduce the inter-observer variation and the burden on clinicians [1, 5–9]. Atlas-based segmentation is a tool that creates a database of contoured CT and automatically delineates for new patients based on that database. However, actual tissues have patterns of varying intensity, making it difficult for computer vision-based algorithms to work effectively in a uniform manner. Recently, researchers have aimed to delineate using artificial intelligence (AI) technology based on deep-learning neural networks (DNNs). AI-based segmentation is a tool to register contoured CT as training data, and delineate for new patients based on the learned network. Many investigations have attempted to develop their in-house AI-based model for application for patients with various cancers for the assessment of delineation accuracy [4, 7–11]. Although these studies state that the AI-based method provides greater accuracy and efficiency than ordinary methods, problems such as required technical skills of development of programing code to implement it and the great effort to collect contoured datasets still remain.

A commercial software has provided advanced technology for AI-based auto-segmentation models. Since this software uses an AI-database on the cloud, there is no necessity to create a database and it is easy to implement. If auto-segmentation accuracy is clinically acceptable, the delineation work can be significantly shortened, and radiotherapy can also be initiated sooner. For rapidly growing tumors, such as head and neck tumors, it is desirable to promptly conduct radiotherapy or often apply adaptive radiotherapy [5].

Our study aims to evaluate the accuracy of an AI-based auto-segmentation model as well as the conventional atlas-based model in comparison with the manual model delineated by radiation oncologists. In this study, the evaluations were performed with efficiency, similarity indices, statistical analysis, and visual evaluation for the patients with prostate cancer or head and neck cancer. This study is the first report to assess atlas- and AI-based delineation accuracy using AI-based auto-segmentation model provided by commercial software that has already been completely trained. By confirming the availability of AI-based model of the commercial software, it is expected to clinically utilize the AI-based segmentation without no effort or time to build the model.

## Methods

### Patients

The auto-segmentation accuracy for the bladder and the rectum for the patients with prostate cancer, the brain stem, mandible, eye, parotid gland, optic chiasm, optic nerve, and spinal cord for the patients with head and neck cancer were evaluated. The prostate was not evaluated because there are not prostate contours in manual delineations at our institution. In this study, target volumes, such as the Gross Tumor Volume or Clinical Target Volume, were not considered. All organs at both sites were manually delineated by three radiation oncologists for patients with prostate cancer, and five for patients with head and neck cancer. The commercial software MIM Maestro (ver. 7.0.3, Cleveland, OH, USA) was used to apply the atlas-based segmentation (SEG_atlas_) and compare the delineations. MIM Contour ProtégéAI (ver. 0.9), which is limited to research use at the time of this study, was used to apply the AI-based segmentation (SEG_AI_). The auto-segmentation accuracies of the two models were evaluated by the comparison with manual delineation as a reference. This study was approved by the institutional review board of the National Cancer Center Hospital in Japan (approval number: 2018–318), and was conducted according to the ethical standards of the Declaration of Helsinki.

### Atlas-based segmentation

A total of 41 patients with prostate cancer treated at our institution were assessed in this study. Among all patients, 20 datasets (CT images with delineations) were registered in the atlas database to build the model. The remaining 21 patients were used for the model evaluation. Similarly, the total number of patients with head and neck cancer was 50. The number of datasets for the model and evaluation were 20 and 30, respectively. The patients’ dataset for modeling was used only in the atlas-based condition. The workstation with 2.5 GHz quad-core processor was used to execute the SEG_atlas_.

To perform SEG_atlas_, the atlas-database was built. The patients for modeling were registered in the MIM database. One patient was selected as the reference patient from among the registered patients. The other registered patients were rigidly aligned to the reference patient to calculate the similarity matrices to the reference patient. Similarity matrices for all registered patients were prepared in advance. As shown in Additional file 1: Figure S1, the workflow of the SEG_atlas_ was as follows: The rigid alignment was performed between the test patient and the reference patient, and the similarity matrix was calculated to search for similar patients from among the registered patients. After identifying several patients with high similarity, the existing structures of the selected patients were propagated to test patients based on the deformable image registration (DIR) [12]. This process was performed several times, and multiple structures were created on test patient's CT image for each site. The final structure was determined by the majority vote method.

The majority vote method in the SEG_atlas_ was used to improve model accuracy. The structures from 5 patients most similar to the test patient were selected from the database and DIR was performed between the 5 patients and the test patient. DIR was performed based on the intensity of CT image. From the propagated 5 delineations, the area in which over 3 delineations overlapped was specified the final contour. For small volume structures such as the eye, optic chiasm, and optic nerve, we adopted one candidate patient whose structure displayed the best matching to the test patient, instead of five, given the small region of overlapping organ volume.

For patients with prostate cancer, the rectum was delineated on the CT images by masking rectal gas to avoid the effect of gas in the rectum. After propagations at both sites, the created atlas-based delineations were subjected to hole-filling, smoothing, and cleaning of tiny fragments of the delineations.

### AI-based segmentation

The AI database on the MIM cloud was used. MIM Contour ProtégéAI is based on neural network for automated delineation on CT and magnetic resonance (MR) images. A neural network constructed of multiple layers is a mathematical model that imitates the network structure of neurons in the human brain [13]. Neural network is commonly used in image recognition by taking advantage of the characteristic of learning from a large amount of data. The neural network model of Contour ProtégéAI is provided on the basis of the U-Net architecture [14]. U-Net is the model for semantic segmentation, which is a method of categorizing each pixel, based on the peripheral pixels. The AI database on the MIM cloud contains approximately 500–1000 registered training data for each treatment site. In the SEG_AI_, 21 patients with prostate cancer and 30 patients with head and neck cancer were evaluated.

### Evaluation of segmentation accuracy

Three similarity metrics were used for evaluation: the Dice similarity coefficient (DSC) [15], Hausdorff distance (HD) [15], and mean distance to agreement (MDA) [16, 17]. The DSC was obtained by dividing the overlapping volume of the two structures, A and B, by the mean volume of the two structures, as shown in the following equation:

1 $$DSC(A,B)=\frac{\left|A\cap B\right|}{\left(\left|A\right|+\left|B\right|\right)/2}$$

When two structures, A and B, matched exactly, the DSC was considered to indicate unity. In this study, the delineation accuracies were determined to be excellent when the DSC value exceeded 0.8.

HD represents the distance between the two structures, denoted as A and B. HD can be calculated using the following equation:

2 $$HD(A,B)=max\left(h\left(A, B\right), h\left(B, A\right)\right)$$

where *h*(*A*, *B*), referred to as the directed HD, given by $h(A,B)={max}_{a\in A}{min}_{b\in B}\Vert a-b\Vert$, where *a* and *b* represent any point at the outlines of structures *A* and *B*, and $\Vert a-b\Vert$ is the Euclidean distance between *a* and *b*. MDA is similar to HD and can be derived by substituting the maximum with mean. If the outlines of the two structures are completely consistent, the HD and MDA approach zero.

The cranial-caudal length of the structures varies in the rectum and spinal cord, and the auto-segmentation accuracies cannot be accurately evaluated. Therefore, the cranial-caudal length in these structures was the same as that of the manual structures created by erasing unnecessary delineations.

In addition, the relative volume errors Δ*V* in the volume of SEG_atlas_ and SEG_AI_ (*V*_auto_) were calculated using the following equation with reference to the volume of the manual delineation (*V*_manual_). In this study, the mean and standard deviation of the relative error were also calculated.

3 $$\Delta V=\frac{\left|{V}_{\mathrm{auto}}-{V}_{\mathrm{manual}}\right|}{{V}_{\mathrm{manual}}}$$

To investigate whether each index of the DSC, HD, and MDA correlated with the volume, the correlation coefficient was calculated. In addition, radiation oncologists visually evaluated the delineation accuracy of SEG_atlas_ and SEG_AI_.

### Statistical analysis

The R ver. 4.0.2 [R Core Team (2016) R: A language and environment for statistical computing. R Foundation for Statistical Computing, Vienna, Austria] was used for statistical analyses. For each index of the DSC, HD, and MDA, the test for independence from volume using Pearson's product moment correlation coefficient was performed with a *p *value < 0.05. For each index of the DSC, HD, and MDA, the two-group difference test was performed with a *p *value < 0.05, for the SEG_atlas_ and the SEG_AI_, respectively. First, the normality of the distribution was confirmed using the Shapiro–Wilk test. In cases of non-normality, the Wilcoxon signed-rank test was performed. When the assumption of normality was met, the variance was tested by the *F*-test, and the Student's *t*-test was performed when the variances of the two samples were equal, and the Welch's *t*-test was performed when the variances were not equal. For comparisons between non-normally and normally distributed samples, the Wilcoxon signed-rank test was performed.

## Results

### Segmentation efficiency

The processing time to create delineations was approximately 3 min per case on the atlas-based model and approximately 5 min (range, 3–10 min) per case on the AI-based model for the patients with prostate cancer. For patients with head and neck cancer, the processing times were both 6 min (range, 3–8 min).

### Prostate cancer patients

#### Evaluation of segmentation accuracy

**Fig. 1:**
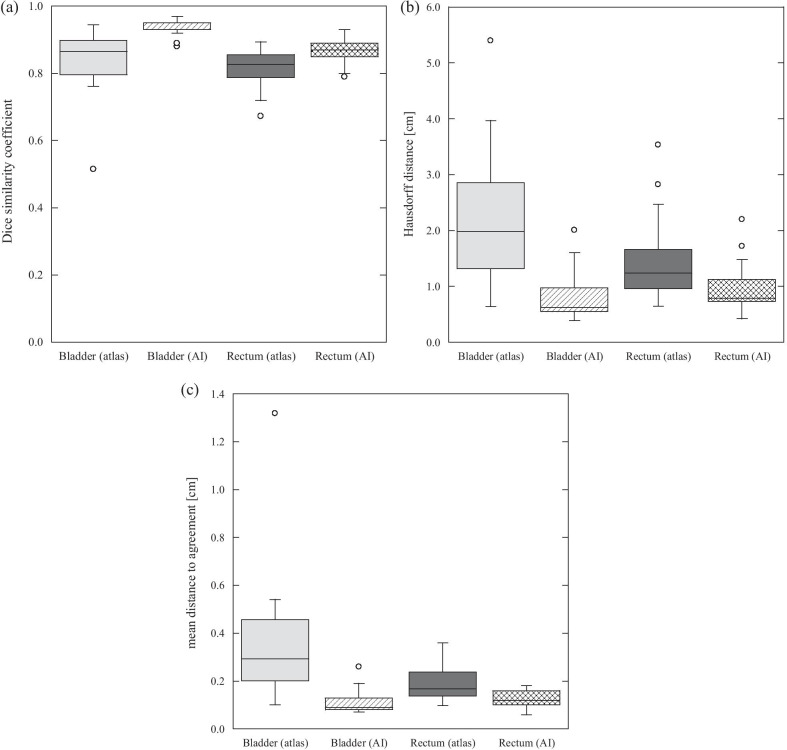
The similarity metrics for the bladder and rectum for SEG_atlas_ (atlas-based segmentation) and SEG_AI_ (AI-based segmentation). **a** Dice similarity coefficient (DSC), **b** Hausdorff distance (HD), **c** mean distance to agreement (MDA)

Figure 1 shows the DSC, HD, and MDA results for the bladder and rectum of patients with prostate cancer. In both the atlas- and AI-based models, the median DSC exceeded 0.8, indicating good agreement with the manual delineations. In the SEG_AI_, the median value was closer to unity, and the interquartile range was smaller than that of the SEG_atlas_. For HD, the median and interquartile range of AI-based assessment was smaller than that of the atlas-based assessment in both the bladder and the rectum. MDA results were similar to those of HD.

In the SEG_atlas_, the mean relative volume error in the bladder was 25.7 ± 35.8%, and the maximum error was 171%. The mean relative volume error for the rectum was 20.9 ± 14.3%, and the maximum error was 47.5%. In the SEG_AI,_ the mean relative volume error for the bladder was 6.2 ± 6.6% and the maximum error was 24.4%. The mean relative volume error for the rectum was 17.6 ± 10.1%, and the maximum error was 49.8%.

We also investigated the relationships between each index of DSC, HD, and MDA, and the volume. Additional file 2: Table S1 shows the Pearson's correlation coefficient *r* between the volume and each index for the bladder and rectum for SEG_atlas_ and SEG_AI_. No significant correlations between all three indexes and the volume were observed (*p* > 0.05).

### Statistical analysis

As shown in Table 1, for each index of DSC, HD, and MDA of the bladder and rectum, two-group difference test was performed using the atlas- and AI-based delineations. All indexes displayed a significant differences between the SEG_atlas_ and SEG_AI_ (*p* < 0.05).

**Table 1:** The *p* values for *t* tests for Dice similarity coefficient (DSC), Hausdorff distance (HD) and mean distance to agreement (MDA) between SEG_atlas_ (atlas-based segmentation) and SEG_AI_ (AI-based segmentation) delineations in patients with prostate and head and neck cancers

	Prostate		Head and neck									
	Bladder	Rectum	Brainstem	Mandible	Eye_L	Eye_R	Chiasma	Optic nerve_L	Optic nerve_R	Parotid_L	Parotid_R	Spinal cord
DSC	0.00	0.00	0.02	0.00	0.70	0.00	0.90	0.14	0.16	0.45	0.10	0.45
HD	0.00	0.02	0.16	0.26	0.02	0.00	0.22	0.97	0.17	0.87	0.04	0.55
MDA	0.00	0.00	0.27	0.00	0.67	0.00	0.48	0.64	0.21	0.62	0.00	0.54

### Visual evaluation

**Fig. 2:**
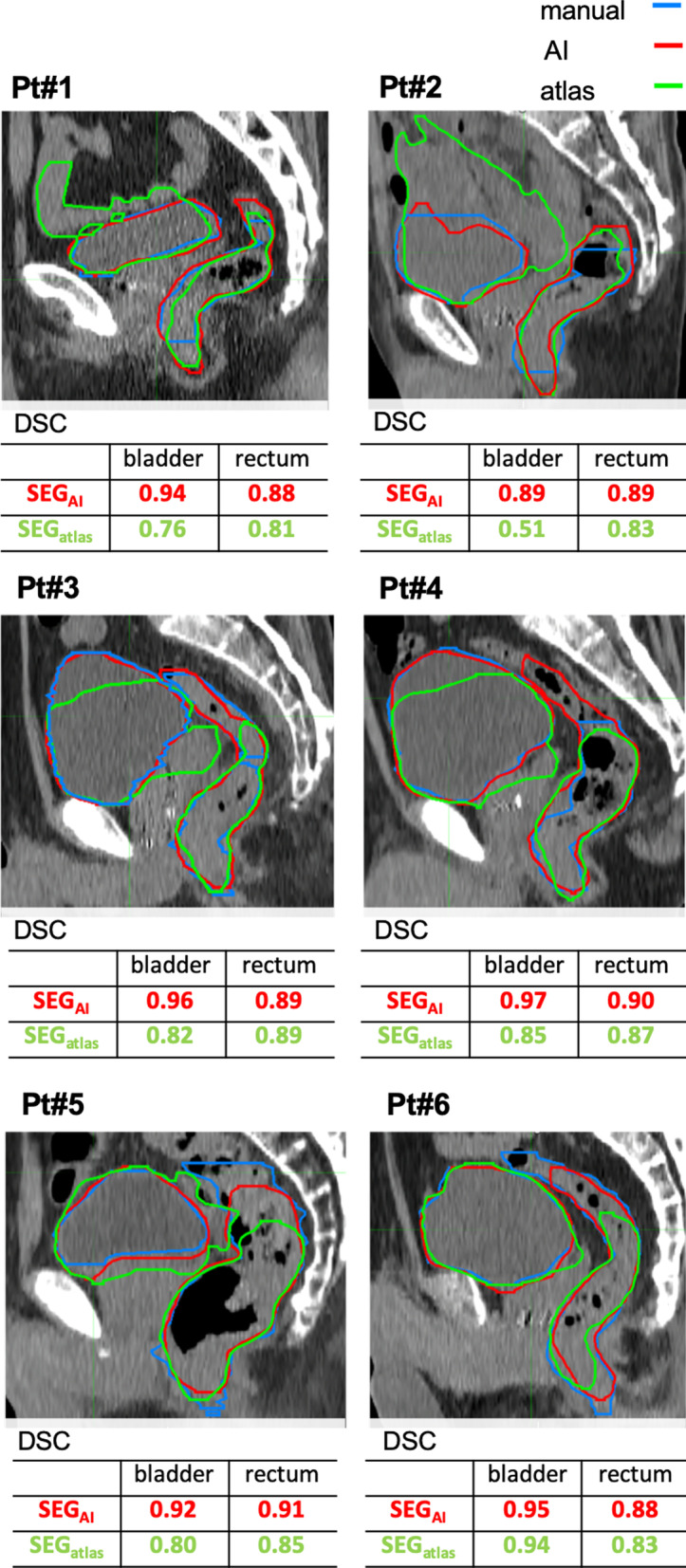
Examples of manual and automatic delineations of the bladder and rectum. SEG_AI_: AI-based segmentation, SEG_atlas_: atlas-based segmentation

Figure 2 shows several examples of SEG_atlas_, SEG_AI_, and manual delineations for the rectum and bladder with indications of the patient number. Some errors in the atlas-based delineation were detected near the boundary when the boundary was unclear, such as the small bowel and the seminal vesicle. On the other hand, the AI-based delineations were more consistent with the manual ones. The surrounding tissue, such as the small bowel, was unexpectedly included with the bladder in 17 of 21 patients in the SEG_atlas_ and 5 of 21 patients in the SEG_AI_.

### Head and neck cancer patients

#### Evaluation of segmentation accuracy

**Fig. 3:**
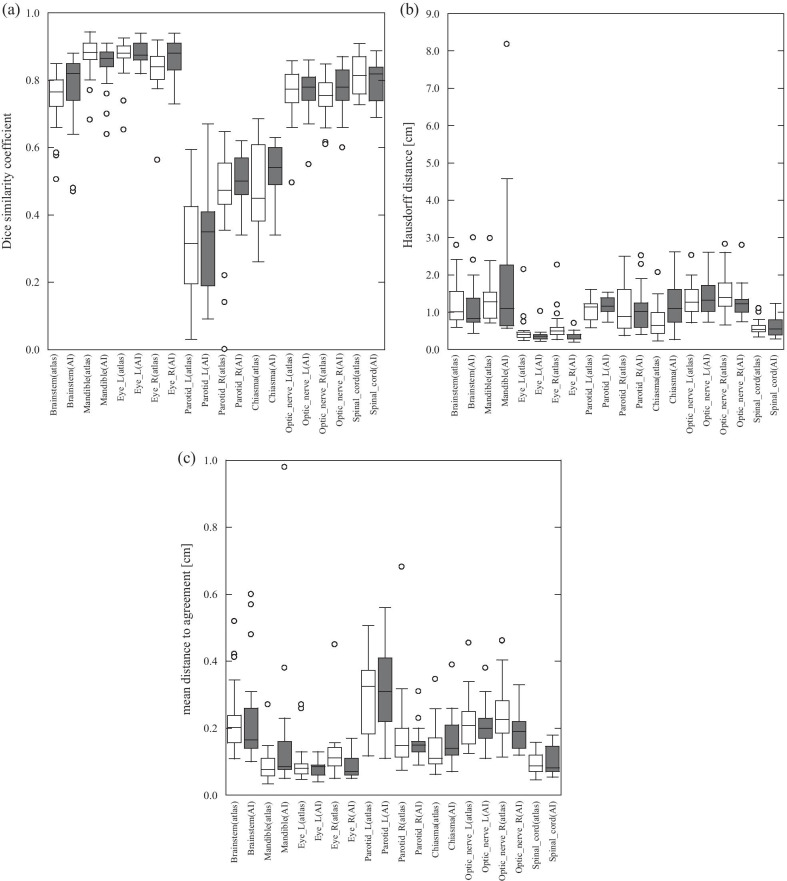
The similarity metrics for each organ of patients with head and neck cancer for SEG_atlas_ (atlas-based segmentation) and SEG_AI_ (AI-based segmentation). **a** Dice similarity coefficient (DCS), **b** Hausdorff distance (HD), **c** mean distance to agreement (MDA)

Figure 3a shows the DSC of OARs for head and neck cancer patients. In the SEG_AI_, the median values of the brain stem, mandible, bilateral eyes, and spinal cord exceeded 0.8. In the SEG_atlas_, the median value exceeded 0.8 for the mandible, bilateral eyes, and spinal cord. However, there were many organs with poor DSC, especially in the optic chiasm and optic nerve, which had a small volume. Figure 3b, c show HD and MDA. In one case, the largest HD was observed for the mandible in the SEG_AI_. The values of the bilateral eyes were close to zero and agreed with the manual delineations. Additionally, the variation in MDA was lower than that in DSC. The interquartile of the DSC for left and right eyes was 0.03 and 0.06, respectively (median 0.88 and 0.84), while the interquartile of MDA was 0.03 and 0.05 (median 0.08 and 0.11), respectively.

The relative volume errors of the SEG_atlas_ and SEG_AI_ were calculated based on the volumes of the manual delineations. Additional file 3: Table S2 shows the relative error in the volume for each organ. In the optic chiasm, the mean error was 72.9% in the atlas-based group and 65.7% in the AI-based group. We also investigated the correlation between each index of DSC, HD, and MDA, and the volume. Additional file 4: Table S3 shows the Pearson's correlation coefficients between the volume and each index.

### Statistical analysis

Table 1 shows each index of DSC, HD, and MDA, and results of two-group difference test performed using the SEG_atlas_ and SEG_AI_ in head and neck cancer patients. Significant differences in all three indices were observed only for the right eye. No significant difference was detected among small tissues, such as the optic chiasm and optic nerve.

### Visual evaluation

**Fig. 4:**
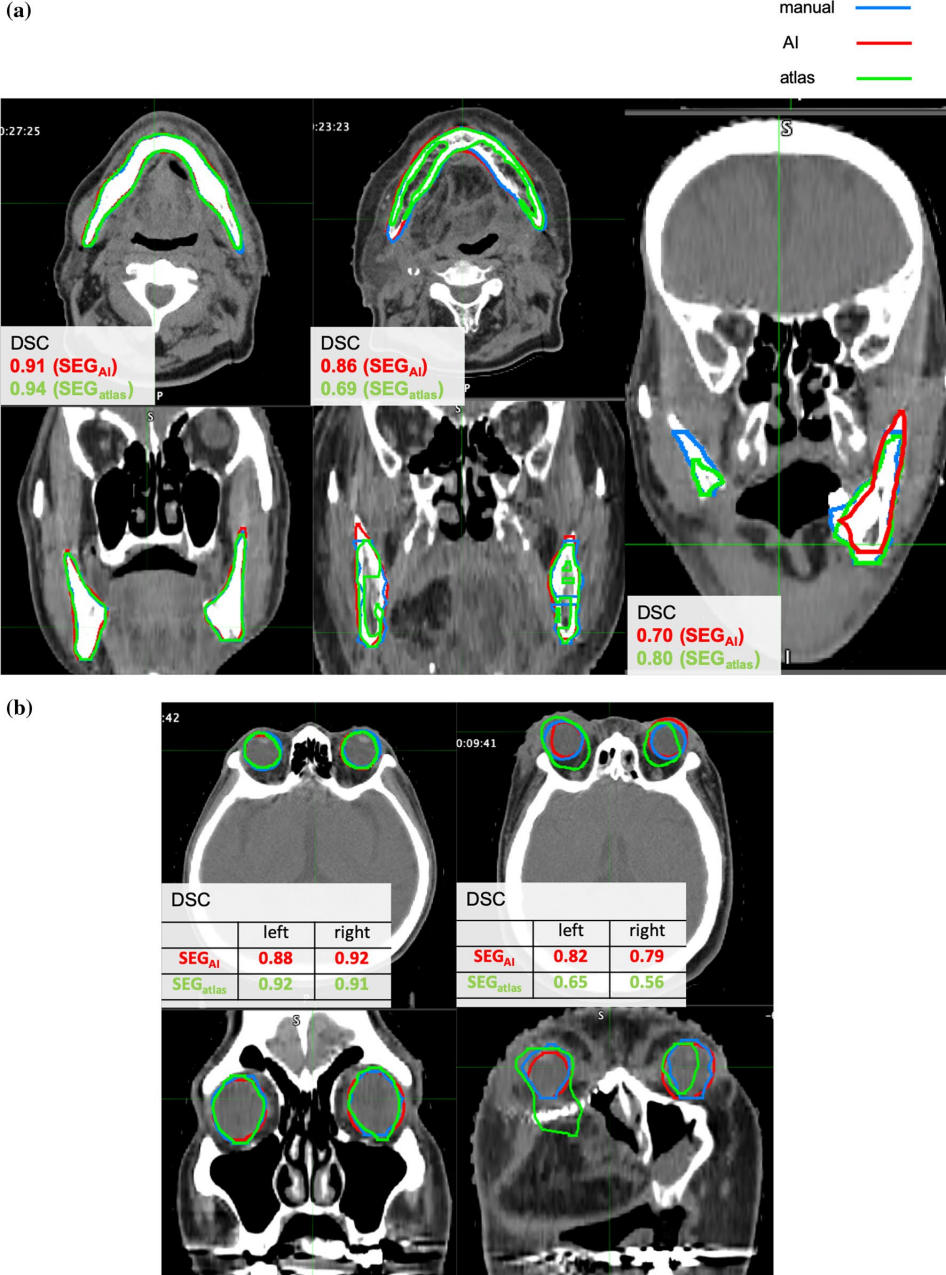

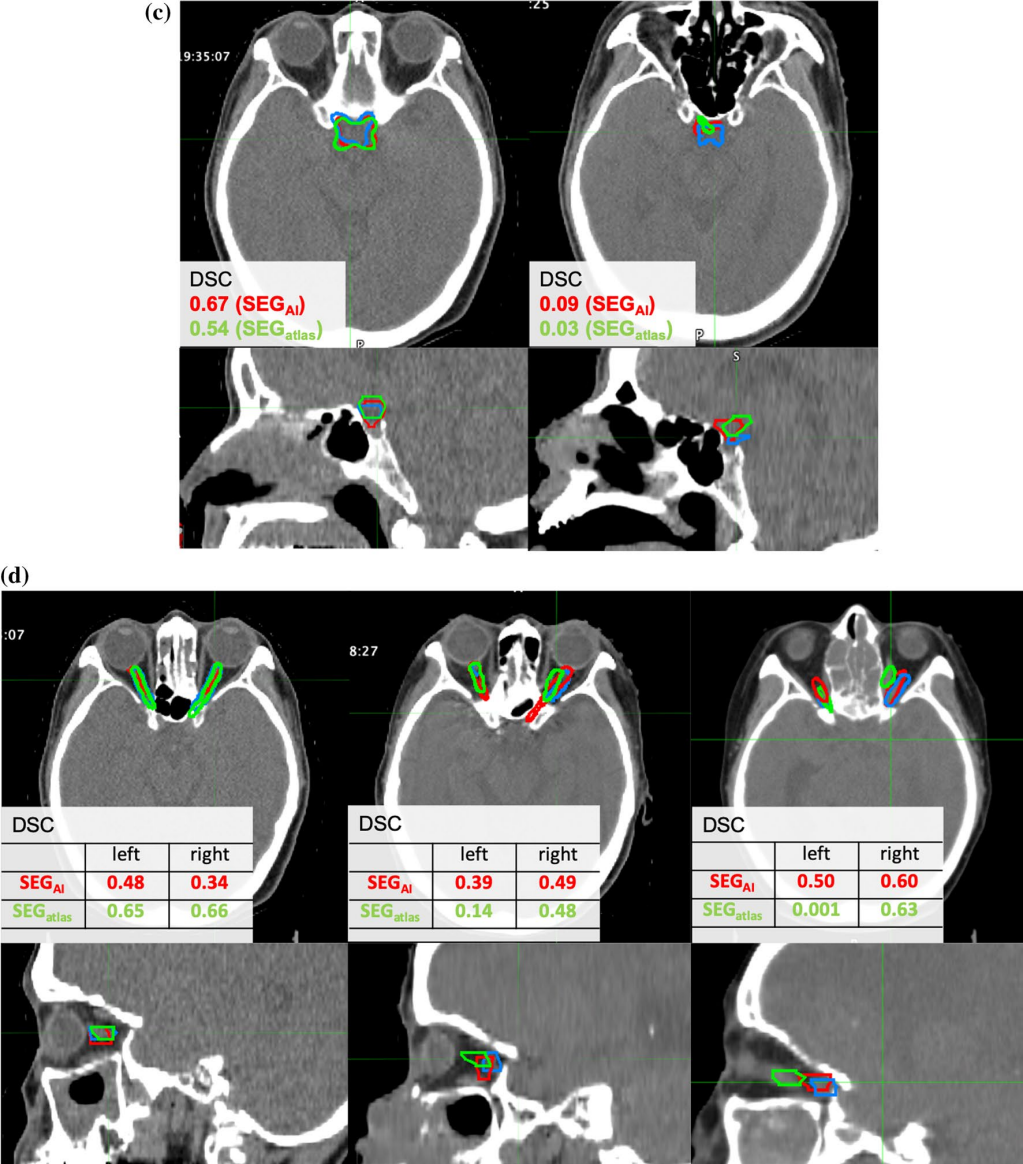
Examples of manual and automatic delineations of the OARs for the head and neck cancer patients. **a** mandible, **b** eyeballs, **c** optic chiasm, **d** optic nerve

Figure 4 shows an example of the SEG_atlas_ and SEG_AI_ and the manual delineations in patients with head and neck cancer, as well as the DSC results. In the mandible, both automatic delineations were very similar to manual delineations in most cases. However, some instances included low DSC in the atlas-based assessment and there was insufficient delineation where there was a cavity within the bone. Additionally, on AI-based assessment, the delineation accuracy for cases after surgical resection of the mandible was inadequate, and this was an outlier in HD and MDA.

Figure 4b shows an example of the delineation of bilateral eyeballs. In many cases of the eyes, automatic delineations were similar to manual ones, but in some cases of atlas-based assessment, the delineation deviated significantly, and the DSC was poor. In the worst case, the DSC was 0.56. The HD and MDA were 2.27 and 0.45 cm, respectively.

Figure 4c shows the delineation of the optic chiasm. Overall, the delineation accuracy was low. In the worst case, the DSC was 0.03. In cases with low DSC, there were many delineations that barely overlapped with the manual ones, even though there was only a slight deviation. The HD and MDA were 1.03 and 0.40 cm, respectively.

Figure 4d shows the delineation of the optic nerve. Overall, the delineation accuracy was low. In the worst case, the DSC was 0.001. Similar to the optic chiasm, there were many delineations that barely overlapped with the manual ones, even though there was only a slight deviation. In the SEG_atlas_, the delineation drawing the muscle around the eye as the optic nerve was observed. The HD and MDA were 1.63 and 0.68, respectively.

## Discussion

There are limited investigations on assessment of accuracy of SEG_AI_, and this topic is still on investigation stages. The fact is that almost all of studies developed in-house software of SEG_AI_ for various target and organs [4, 7–11]. Recently, commercial AI-based model became available, and we aimed to evaluate the AI-based model provided by the commercial software. Commercial AI-based model, compared with in-house AI-based model, features reduction of effort and time on the training data collection and model construction. Our study provides useful information on the commission of a commercial AI-based model for clinical implementation.

The delineations of the bladder and rectum using AI-based model was more accurate in all three indexes of DSC, HD, and MDA (Fig. 1). For instance, Fig. 1b shows that the difference in median HD for the two models was as large as 1.37 cm for the bladder and 0.45 cm for the rectum. In addition, the results of SEG_atlas_ tended to have large outliers and all organs exhibited the interquartile percentile being larger than that of SEG_AI_ (Fig. 1). Among the three indexes, significant differences were detected between SEG_atlas_ and SEG_AI_. For visual evaluations, there were some errors in the atlas-based delineation near the boundary, particularly when the boundary was unclear, such as near the small bowel and seminal vesicle (Fig. 2). The AI-based delineations were relatively similar to manual delineations. For the rectum, many atlas-based delineations were significantly smaller than the manual ones. This is because the segmentation was performed by the majority vote method, which adopts the overlapping region of the objects of multiple candidate patients’ models. Although visual evaluation revealed that both atlas- and AI-based delineations require manual correction, fewer correction-required-SEG_AI_ indicates the segmentation efficiency of AI-based model is higher than that of atlas-based model.

There were no significant differences in similarity indexes between the SEG_atlas_ and SEG_AI_ in head and neck, which was inconsistent with the results of Wen et al. [9] who evaluated four mastication muscles. Their results demonstrated that AI-models outperformed atlas-model. However, we evaluated various common head and neck organs, and the size and the form of organs, and CT-values varied. Of all organs evaluated in this study, only the brainstem demonstrated that the AI-model was closer to 1 for DSC and closer to 0 for HD or MDA, while there was no significant difference (HD: *p* = 0.16, MDA: *p* = 0.27). Except for the right eye, all other organs displayed no significant differences, therefore, accuracy of the two models was comparable.

Regarding DSC (Fig. 3a, b), both SEG_atlas_ and SEG_AI_ displayed high segmentation accuracy in the mandible and eye. However, low accuracies in small volume organs, such as the optic nerve and optic chiasm, were observed. Alternatively, the HD demonstrated comparable or better results in such organs (Fig. 3c, d), due to differences in the derivation of the two methods. The DSC is calculated from the overlapping of the two delineations and HD is calculated from the simple distance between the two delineations. In the case of small volume delineation, the DSC tends to be small, even if the displacement of the two delineations is acceptable. This suggests that not only DSC, but also HD or MDA, should be used for evaluation, and utilizing only one index should be avoided. When evaluating the delineations, especially for small volumes, it is recommended to use multiple indexes properly and perform visual checks for evaluation.

The advantages and disadvantages of SEG_atlas_ and SEG_AI_ are described as follows: In the SEG_AI_, the delineations are created on the cloud-based system and downloaded in the Digital Imaging and Communication in Medicine (DICOM) format, Radiotherapy (RT) Structure, the efficiency of SEG_AI_ is, therefore, dependent on the internet environment rather than the processing capacity of a workstation. Additionally, a wide variety of delineations can be created because a large amount of training data are registered in the AI database. However, since training data from all over the world are registered on the cloud, data regarding the registered patients’ characteristics are unavailable. The registered dataset may include a variety of patient characteristics. Therefore, the influence of variations in physiques between races remains unclear. Moreover, other institutional characteristics of delineation may be reflected. For instance, the deviation between the delineations for the femur heads and prostate among institutions is possibly caused by reference to a delineation guideline or each institution's own protocol. In addition, the delineation accuracy for the bladder can be associated with bladder volume. If different bladder management protocols are included in the registered patients, delineation accuracy may probably worsen. Actually, a full bladder is commonly recommended for prostate cancer patients, and the effect associated with bladder volume is smaller. Since full bladder management was applied in this study, the DSC of the bladder was 0.95, and it agreed with manual delineation. Notably, it should be validated whether the delineations by the AI-based model satisfy the institution's own protocol prior to clinical implementation. Otherwise, if there is an overemphasis on delineation efficiency and an AI-based model is clinically implemented without the validation process presented in this study, it may unexpectedly promote deterioration of not only standardization of delineations, but also quality of treatment. The SEG_atlas_ requires more time to create delineations than SEG_AI_, and it is dependent on the specifications of the workstation. However, the SEG_atlas_ can reflect institutional delineation protocols, because that institution’s patients can be registered with the modeling data. Registration of a large amount of modeling data to achieve efficient accuracy is necessary prior to use. These two models have some advantages and disadvantages. The automatic segmentation method should be considered according to the purpose, institutional delineation protocol, and working environment.

The limitations of this study were that only the OARs were included, and the Gross Tumor Volume or Clinical Target Volume was not investigated. Although the MIM Contour ProtégéAI can automatically delineate target and normal tissues, target was not a focus of this study. Twenty datasets were used to build the atlas database in this study. The MIM Maestro recommends registering over 20 datasets for the atlas-based model, however, the effect according to the number of datasets is unclear. Thus, improvement of delineation accuracy by increasing the number of datasets requires further investigation. The delineation accuracies were evaluated using only similarity indices and visual evaluation, and the dosimetric impact of segmentation inaccuracy remains unclear. In the future, we plan to assess delineation of other organs and targets. We also plan to perform MRI-based automatic segmentation.

## Conclusions

The atlas- and AI-based delineation accuracies for OARs for patients with prostate and head and neck cancers were evaluated with manual delineations performed by radiation oncologists. SEG_atlas_ and SEG_AI_ are comparable of efficiency. The costs in time for both automatic delineations methods are six minutes on the head and neck cases which is much shorter than the three hours required for manual delineation. On the other hand, on the quantitative evaluation using DSC, HD and MDA, higher accuracies were shown in SEG_AI_ for prostate cancer while no significant difference is shown for head and neck cancer between these two models. According to the results of visual evaluation, even both atlas- and AI-based require manual correction, less necessity of correction in SEG_AI_ indicates that the segmentation efficiency of AI-based model is higher than that of atlas-based model. In conclusion, the effectiveness of the commercial AI-based model can be expected to improve the segmentation efficiency and to significantly shorten the delineation time.

## Supplementary Information

**Additional file 1**. **Supplementary Figure 1:** The workflow of the atlas-based segmentation.

**Additional file 2**. **Supplementary Table 1:** The Pearson's correlation coefficient r of volume and metrics in the bladder and rectum for the atlas-based segmentation and the AI-based segmentation.

**Additional file 3**. **Supplementary Table 2:** The relative error between manual and automatic delineation volume at each organ.

**Additional file 4**. **Supplementary Table 3:** The Pearson's correlation coefficient between three similarity indexes and volumes at each site of head and neck cancer patients.

## Data Availability

All data generated or analyzed during this study are included in this published article.

## Abbreviations

CT: Computed tomography
MR: Magnetic resonance
TPS: Treatment planning system
IMRT: Intensity-modulated radiotherapy
OARs: Organs at Risk
DSC: Dice similarity coefficient
AI: Artificial intelligence
DNN: Deep-learning neural network
SEG_atlas_: Atlas-based segmentation model
SEG_AI_: AI-based segmentation model
DIR: Deformable image registration
HD: Hausdorff distance
MDA: Mean distance to agreement
DICOM: Digital Imaging and Communication in Medicine
RT: Radiotherapy
