# Supplementary figures and images for: Evaluation of auto-segmentation accuracy of cloud-based artificial intelligence and atlas-based models
Source: Radiat Oncol. 2021 Sep 9;16:175. doi: 10.1186/s13014-021-01896-1 (PMC8427857; doi:10.1186/s13014-021-01896-1)

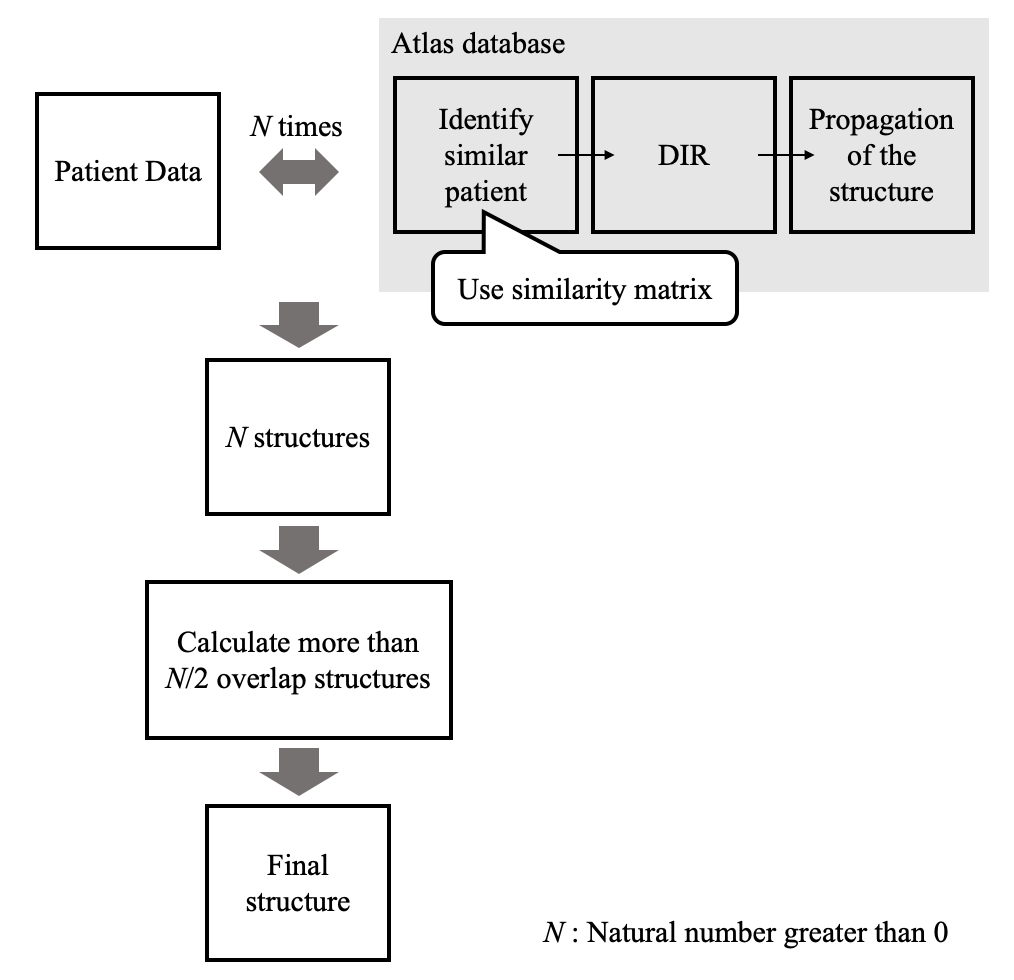


Supplementary Figure 1. The workflow of the SEGatlas (atlas-based segmentation)

Supplement: Supplementary file 1 — Additional file 1. Supplementary Figure 1: The workflow of the atlas-based segmentation. [file 13014_2021_1896_MOESM1_ESM.docx]
