# Supplementary material for: Evaluation of auto-segmentation accuracy of cloud-based artificial intelligence and atlas-based models
Source: Radiat Oncol. 2021 Sep 9;16:175. doi: 10.1186/s13014-021-01896-1 (PMC8427857; doi:10.1186/s13014-021-01896-1)
Supplement: Supplementary file 2 — Additional file 2. Supplementary Table 1: The Pearson's correlation coefficient r of volume and metrics in the bladder and rectum for the atlas-based segmentation and the AI-based segmentation. [file 13014_2021_1896_MOESM2_ESM.docx]

Supplementary Table 1. The Pearson's correlation coefficient *r* of volume and metrics in the bladder and rectum for SEG_atlas_ (atlas-based segmentation) and SEG_AI_ (AI-based segmentation)

|  | | SEG_atlas_ | SEG_AI_ |
| --- | --- | --- | --- |
|  |  | *r* | |
| DSC  (Dice similarity coefficient) | Bladder | 0.36  *p* = 0.10 | 0.36  *p* = 0.11 |
|  | Rectum | -0.01  *p* = 0.98 | 0.30  *p* = 0.18 |
| HD  (Hausdorff distance) | Bladder | -0.32  *p* = 0.16 | 0.02  *p* = 0.92 |
|  | Rectum | 0.21  *p* = 0.35 | 0.29  *p* = 0.20 |
| MDA  (mean distance to agreement) | Bladder | -0.29  *p* = 0.20 | -0.00  *p* = 0.99 |
|  | rectum | 0.33  *p* = 0.14 | 0.09  *p* = 0.69 |
