# Supplementary material for: Evaluation of auto-segmentation accuracy of cloud-based artificial intelligence and atlas-based models
Source: Radiat Oncol. 2021 Sep 9;16:175. doi: 10.1186/s13014-021-01896-1 (PMC8427857; doi:10.1186/s13014-021-01896-1)
Supplement: Supplementary file 3 — Additional file 3. Supplementary Table 2: The relative error between manual and automatic delineation volume at each organ. [file 13014_2021_1896_MOESM3_ESM.docx]

Supplementary Table 2. The relative error between manual and automatic delineation volume at each organ

|  | Bladder | Rectum | Brainstem | Mandible | Eye_L | Eye_R | Chiasma | Optic nerve_L | Optic nerve_R | Parotid_L | Parotid_R | Spinal cord |
| --- | --- | --- | --- | --- | --- | --- | --- | --- | --- | --- | --- | --- |
| SEG_atlas_ (atlas-based segmentation) | | | | | | | | | | | | |
| Mean ± SD [%] | 25.7±35.8 | 20.9±14.3 | 25.2±11.9 | 12.1±13.3 | 13.6±14.5 | 30.1±31.4 | 72.9±55.6 | 24.9±14.7 | 32.0±14.2 | 30.7±39.2 | 22.2±21.7 | 31.6±22.2 |
| Maximum [%] | 170.9 | 47.5 | 58.3 | 47.9 | 58.5 | 150.0 | 225.8 | 53.6 | 73.0 | 185.1 | 78.6 | 64.8 |
| SEG_AI_ (AI-based segmentation) | | | | | | | | | | | | |
| Mean ± SD [%] | 6.2±6.6 | 17.6±10.1 | 19.1±22.4 | 11.2±11.7 | 12.0±9.8 | 15.2±11.5 | 65.7±43.3 | 25.2±19.9 | 33.4±20.4 | 20.3±24.5 | 20.3±14.2 | 23.6±14.3 |
| Maximum [%] | 24.4 | 49.8 | 85.3 | 49.7 | 30.1 | 38.9 | 168.2 | 69.3 | 81.6 | 121.7 | 58.1 | 47.2 |
