# Supplementary material for: Evaluation of auto-segmentation accuracy of cloud-based artificial intelligence and atlas-based models
Source: Radiat Oncol. 2021 Sep 9;16:175. doi: 10.1186/s13014-021-01896-1 (PMC8427857; doi:10.1186/s13014-021-01896-1)
Supplement: Supplementary file 4 — Additional file 4. Supplementary Table 3: The Pearson's correlation coefficient between three similarity indexes and volumes at each site of head and neck cancer patients. [file 13014_2021_1896_MOESM4_ESM.docx]

Supplementary Table 3. The Pearson's correlation coefficient between three similarity indexes and volumes at each site of head and neck cancer patients

|  | Brainstem | Mandible | Eye_L | Eye_R | Chiasma | Optic nerve_L | Optic nerve_R | Parotid_L | Parotid_R | Spinal cord |
| --- | --- | --- | --- | --- | --- | --- | --- | --- | --- | --- |
| SEG_atlas_ (atlas-based segmentation) | | | | | | | | | | |
| DSC | -0.12  *p* = 0.54 | 0.32  *p* = 0.14 | 0.25  *p* = 0.32 | 0.18  *p* = 0.47 | -0.29  *p* = 0.23 | -0.40  *p* = 0.10 | -0.16  *p* = 0.55 | 0.55  *p* = 0.01 | 0.28  *p* = 0.20 | 0.79  *p* = 0.00 |
| HD | -0.08  *p* = 0.68 | -0.13  *p* = 0.56 | -0.20  *p* = 0.43 | -0.20  *p* = 0.42 | 0.57  *p* = 0.01 | -0.01  *p* = 0.96 | 0.13  *p* = 0.61 | 0.23  *p* = 0.29 | 0.29  *p* = 0.18 | 0.20  *p* = 0.30 |
| MDA | 0.10  *p* = 0.61 | -0.11  *p* = 0.61 | -0.23  *p* = 0.36 | -0.20  *p* = 0.41 | 0.63  *p* = 0.00 | 0.19  *p* = 0.45 | 0.14  *p* = 0.59 | -0.27  *p* = 0.21 | 0.09  *p* = 0.69 | -0.72  *p* = 0.00 |
| SEG_AI_ (AI-based segmentation) | | | | | | | | | | |
| DSC | 0.41  *p* = 0.03 | 0.75  *p* = 0.00 | 0.50  *p* = 0.04 | 0.26  *p* = 0.28 | -0.01  *p* = 0.96 | -0.16  *p* = 0.52 | -0.01  *p* = 0.98 | 0.55  *p* = 0.01 | 0.39  *p* = 0.07 | -0.65  *p* = 0.00 |
| HD | -0.30  *p* = 0.13 | -0.62  *p* = 0.00 | -0.38  *p* = 0.12 | -0.05  *p* = 0.84 | 0.32  *p* = 0.19 | 0.05  *p* = 0.83 | 0.00  *p* = 0.99 | 0.08  *p* = 0.71 | 0.16  *p* = 0.46 | 0.69  *p* = 0.00 |
| MDA | -0.32  *p* = 0.10 | -0.62  *p* = 0.00 | -0.42  *p* = 0.08 | -0.25  *p* = 0.29 | 0.64  *p* = 0.00 | 0.18  *p* = 0.48 | 0.13  *p* = 0.62 | -0.23  *p* = 0.29 | -0.07  *p* = 0.74 | 0.68  *p* = 0.00 |
